# Freshwater Lacustrine Zooplankton and Microplastic: An Issue to Be Still Explored

**DOI:** 10.3390/toxics11121017

**Published:** 2023-12-13

**Authors:** Jassica Lawrence, Carlotta Santolini, Gilberto Binda, Stefano Carnati, Ginevra Boldrocchi, Andrea Pozzi, Roberta Bettinetti

**Affiliations:** 1 DISAT Department of Science and High Technology, University of Insubria, Via Valleggio 11, 22100 Como, Italy; jlawrence@uninsubria.it (J.L.); carlotta.santolini@iusspavia.it (C.S.); scarnati@uninsubria.it (S.C.); andrea.pozzi@uninsubria.it (A.P.); 2 University School for Advanced Studies IUSS, 27100 Pavia, Italy; 3 Norwegian Institute for Water Research, Økernveien 94, 0579 Oslo, Norway; 4 DiSUIT Department of Human Science and Innovation for the Territory, University of Insubria, Via Valleggio 11, 22100 Como, Italy; ginevra.boldrocchi@uninsubria.it

**Keywords:** microplastic, lake, freshwater, zooplankton

## Abstract

Lakes are essentially interlinked to humans as they provide water for drinking, agriculture, industrial and domestic purposes. The upsurge of plastic usage, its persistence, and potential detrimental effects on organisms cause impacts on the trophic food web of freshwater ecosystems; this issue, however, still needs to be explored. Zooplankton worldwide is commonly studied as an indicator of environmental risk in aquatic ecosystems for several pollutants. The aim of the review is to link the existing knowledge of microplastic pollution in zooplankton to assess the potential risks linked to these organisms which are at the first level of the lacustrine trophic web. A database search was conducted through the main databases to gather the relevant literature over the course of time. The sensitivity of zooplankton organisms is evident from laboratory studies, whereas several knowledge gaps exist in the understanding of mechanisms causing toxicity. This review also highlights insufficient data on field studies hampering the understanding of the pollution extent in lakes, as well as unclear trends on ecosystem–level cascading effects of microplastics (MPs) and mechanisms of toxicity (especially in combination with other pollutants). Therefore, this review provides insight into understanding the overlooked issues of microplastic in lake ecosystems to gain an accurate ecological risk assessment.

## 1. Introduction

Lakes play a vital role in providing ecosystem services [1]. However, human activities are impacting these fundamental ecosystems. Here, zooplankton is a group of invertebrate organisms which represents one of the key communities for ecosystem functioning: it plays a pivotal role as a mediator “organism” in food webs, promoting exchange between primary producers (phytoplankton) and the upper levels (invertebrate predators), and is reactive to changes in abiotic factors through space and time [2,3,4]. Freshwater zooplankton is particularly sensitive to anthropogenic activities, such as land use change and urbanization [5,6,7,8], and is commonly studied worldwide [3,9,10,11]. Ecotoxicology research focused mostly on pollution caused by chemicals to zooplankton functionality and ecology in freshwater ecosystems [12,13,14,15], while plastics still represent an unexplored issue [16].

Plastic has emerged as a potential major disturbance of freshwater ecosystems at a global scale [17]: the yearly plastic production rate was around 370 million metric tons in 2019 [18] and almost 4.6% goes into marine waters through rivers and lakes [19,20]. In freshwater ecosystems, the common plastic constituents are polyethylene (PE) and polypropylene (PP), followed by polystyrene (PS), polyvinyl chloride (PVC), polyamide (PA) and polyethylene terephthalate (PET) [21]. Large plastic parts break down in microplastics (<5 mm in size—MPs), which can more easily enter the food web [22]. Moreover, plastics constitute a number of chemical compositions [23] and are capable of adsorption of organic pollutants present in the surrounding media [24]. Since these compounds are carried to organisms by ingestion, microplastics may serve as vectors for organic and inorganic contaminants [25] and expose biodiversity to these harmful chemicals [26,27].

While the presence of MPs is common in both marine and freshwater ecosystems, emphasis has been given firstly on marine water: the surging of microplastics in fresh water is therefore a critical matter to examine [28].

**Figure 1:**
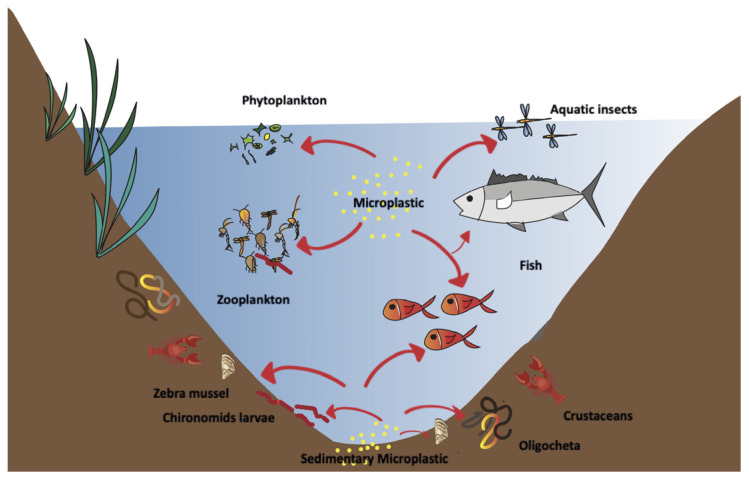
Scheme of MPs uptake route and potential transfer in the trophic web of the lake ecosystem.

Microplastics possess bioaccumulation capability [29], which can increase at diminishing particle size [30,31], making them easily transferable in the trophic web [32,33] during prey–predator collaboration, directly or indirectly [34]. The aquatic organisms from plankton and fish to birds and even mammals in the ecosystem may accumulate microplastics in the food web [22] (Figure 1). Few studies have been conducted on microplastics in freshwater lakes as more work is done on the marine environment. Studies accountable for fresh water and microplastics are estimated to be less than 4% [35,36,37,38]. Based on this limitation, it was found that a conspicuous fraction of MPs are found in fresh water [39] following a heterogenous distribution pattern [40]: this is due to abundant land–based sources, combined with easy transport routes and other non–point sources of MPs (e.g., atmospheric deposition) [41,42]. Thus, the understanding of MPs pollution and the evaluation of the effects on zooplankton in lakes can be a key sentinel of ecosystem–level impacts of MPs in freshwater bodies, as zooplankton is the first step of the trophic web in lakes. Therefore, the aim of the present review is to analyze the state of the art concerning lacustrine zooplankton and MPs.

## 2. Database Search for Articles on Microplastics and Freshwater Zooplankton

The search was conducted using the “Web of Science”, “Scopus”, and “Google Scholar” databases, employing keywords such as microplastic, zooplankton, freshwater, lakes. Additionally, another search was performed with keywords including microplastic, zooplankton, laboratory experiments, freshwater. The research outputs obtained in all the search engines were merged and combined. Subsequently, the research was refined by merging the results related to lakes and laboratory experiments conducted with lacustrine zooplankton. Based on the aim of the review, exclusivity criteria were applied to retain the precision of the review. Articles relating to marine environments, wastewater studies, modeling approaches, reviews encompassing other than freshwater bodies, and those focusing on biota excluding zooplankton were systematically excluded. Therefore, as a result, a total of 49 articles were selected, covering the period from 2016 to the present (2023), indicating a slight increase in the number of articles over time.

## 3. Zooplankton and Microplastics in Lab Studies: Assessing Hazard and Exposure of Microplastics

### 3.1. Effects to Individuals and Different Endpoints

The use of zooplankton species is common to assess the exposure to several pollutants, including MPs (Table 1 specifies all the results discussed in the following paragraphs). Zooplankton organisms, (e.g., *Daphnia* spp., copepods, rotifers) are found to be highly sensitive towards microplastics, especially considering several secondary endpoints such as motility, morphology, reproduction, pulsation, digestion processes and oxidative stress [43,44].

Interestingly, De Felice et al. [45] exposed zooplankton for 21 days (chronic test) to polystyrene (PEST) nanoplastics, observing no effects on oxidative stress and swimming activity, even if a noticeable alteration in energy preservation was developed. Behavioral alterations upon the exposure of zooplankton to PS microplastics such as changes in swimming and phototactic locomotion were also observed by De Felice et al. [46].

Whereas, Tang et al. [47] conducted the experiment of MPs and their impacts on the DNA of zooplankton for three concentrations of MPs for 10 days and observed that oxidative mechanism, energy formation and cellular transfer activities were increased noticeably at 2 and 4 mg/L while at 8 mg/L the progress was declined. Aljaibachi et al. [48] investigated the intake, detention, and the effects of 2 μm PS MPs in *D. magna* with respect to food ingestion with *Chlorella vulgaris*, in the presence of algae; microplastic consumption was reduced, indicating that *Daphnia* did not conveniently eat algae. The mature *Daphnia* showed mortality after 7 days with 21 days of exposure, but reproduction was not impacted. Chen et al. [49] detected the effects of colored microplastics and algae on *D. magna* feeding characteristics. It was revealed that *Daphnids* were unable to differentiate between colored MPs and algae. However, an interesting observation was found that algae consumption increased as MPs were 40% of algal cells, possibly due to *daphnids* maybe broadening their filtering gapes with diminishing food quality. The process was stopped shortly as the result of flocculation of MPs and algae settled down the *Daphnia*.

### 3.2. Role of Particle Characteristic in Toxicity

As with other particulate matters in water, the particle size and shape, the surface properties and chemistry of MPs may play a role in their exposure route and, in turn, in their toxicity to zooplankton. In the following subsections, we review the effects of particle properties and chemicals observed in exposure studies on zooplankton.

#### 3.2.1. Role of Particle Size

**Figure 2:**
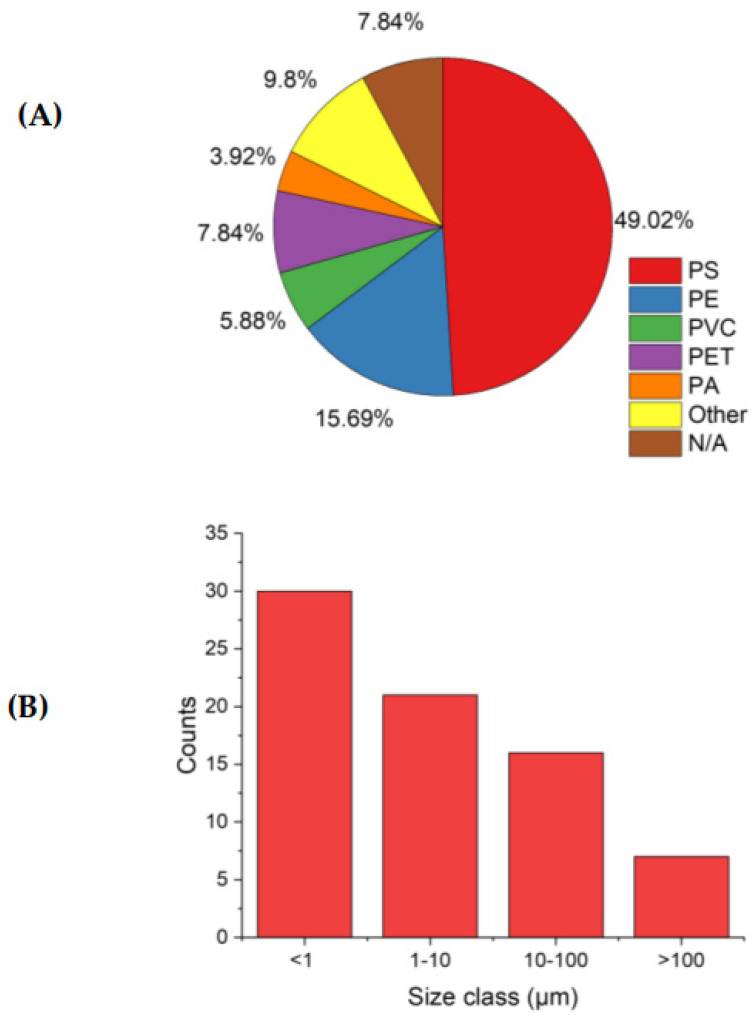
(**A**) Pie chart showing the polymers used in different ecotoxicological tests to expose zooplankton species. N/A indicate the studies where polymer type was not defined. (**B**) Average size distribution of the MPs used in the ecotoxicological tests reviewed here.

An important factor in defining the exposure of MPs to zooplankton is the particle size. The particle size, for example, is key to define the potential exposure by particle ingestion to zooplankton: for *D. magna*, the maximum estimated ingestible size is 114.87 μm [87]. The size of particles also defines the probability of transmovement in tissues leading to inflammation. Koelmans et al. [87] generally stated that the standard MPs size range for this process is between 10 nm and 3 μm. Considering the size of MPs used in the studies reviewed in this paper (Figure 2), it is evident that the main uptake route analyzed so far included the ingestion of fragments by organisms, while a minor component of studies assessed the effects also of MPs with coarser grain size. In addition, the studies assessing the effects generally analyze concentrations of MPs which are generally up to several orders of magnitude higher than the environmental ones [88].

Zhu et al. [50] analyzed PS–MPs gathered in the digestive tract of zooplankton. It was found that 2 and 4 mg/L of PS–MPs caused inhibition in endurance capabilities. The 21– or 14–day exposure up to 4 mg/L of MPs constrained the body length and reproduction. Also, contact of 500 nm PS–MPs for 14 days constrained glucose, and fructose constituents consequently perturbed the system of lipid transfer and exertion. Interestingly, PS–MPs triggered DNA restoration but retarded the lipid ingestion. The smaller–size particles caused severe toxicity, and similar trends were observed with long–term contact to large–size 500 nm particles, including restriction in energy and antioxidant catabolism. Kokalj et al. [51] analyzed four different MPs from two facial cleansers, a plastic bag and PE textile fleece. The mean size range of the particles was 20 to 250 μm, and it was found that four of them were in the guts of *D. magna*. MPs below 100 μm were taken up in the gut. The increase in size of MPs led to a reduction in the gut. The exposure was not hazardous to *D. magna*. The size of MPs clearly impacts the potential exposure of zooplankton: as an example, Rehse et al. [52] investigated the short–term exposure of MPs using PE particles at sizes between 1 μm and 100 μm up to 96 h. Only the 1 μm particle size was ingested and caused movement restriction with prolonged exposure and dose, whereas 100 μm was not absorbed and did not have impacts.

Jeong et al. [53], for example, found increased uptake of MPs of smaller size (0.05 μm) as compared to micro–sized beads (6 μm), leading to oxidation stress due to lipid membranes’ deterioration. Mao et al. [54] evaluated instead the individual effects and combined effects of microplastic on zooplankton. The results revealed that small–size MPs have harmful effects on the life period, hatching time of eggs, population growth and fertility as compared to large–size MPs. Moreover, the synergistic effects of varied–size MPs with other pollutants on the life period, reproduction and population growth were noticeable. Likewise, Rosenkranz et al. [55] compared two different size microplastics (i.e., 20 nm and 1000 nm average particle size) on zooplankton. Fast collection of both particles was observed in the digestive tract within an hour of contact. The 20 nm particles were low with respect to mass but were equivalent to 1000 nm particles if the total surface area is considered. Defecation was fast compared to 1000 nm particle uptake, declining by more than 90% over 4 h. Comparatively, defecation of 20 nm was slow, almost 40% over 4 h.

Similarly, De Felice et al. [46] investigated the effects of microplastic absorption and desorption on the behavior of zooplankton for 21 days with two differently sized PS microplastic 1 and 10 μm on zooplankton and discovered that fecundity, phototactic habits and swimming abilities were affected. Both particles remained in the digestive tract of zooplankton beyond 96 h. As compared to most of the studies, this study revealed an enhancement in body development, swimming ability, amplified brood despite the contact with the highest microplastic size. Moreover, negative impacts were observed on population tendencies. Jeong et al. [56] detected the negative impacts of ingestion and egestion MPs exposure of size 0.05, 0.5 and 6 μm nonfunctionalized PS microbeads. The results revealed that PS microbeads led to the declined development, reproduction, with consequences on life period and delay in reproduction. Moreover, 6 μm microbeads were removed more easily than the other sizes of microbeads, clearly suggesting size–dependent effects.

#### 3.2.2. Role of Morphology and Particle Chemical Features

Chemical properties of MPs can also play an important role in defining their toxicity, as well as the set of additives often included in formulates [89,90]. Generally, the polymer type is the main variable assessed to observe different responses to MPs exposure. Zimmermann et al. [57] analyzed three different types of uneven microplastics, PVC, polyurethane (PUR) and polylactic acid (PLA) MPs on zooplankton for 21 days. The three plastics showed a negative impact on the life history of zooplankton. PVC adversely impacts fecundity. PLA declined the endurance abilities. However, it is worth considering that the most frequently used polymer in ecotoxicological tests (in almost 50% of the analyzed studies) is PS.

Rosenkranz et al. [55] also evaluated the effect of plastic surface properties (such as rugosity, surface area) and the effects of plastic ageing (i.e., the biotic or abiotic degradation of the polymer structure) which alters the surface properties and the effects of MPs on zooplankton. An et al. [58] investigated the effects of MPs beads and fragments on *D. magna* for 21 days. The contact of MPs fragments showed a noticeable decline in endurance compared to contact with MPs beads. The endurance capability of *D. magna* in contact with small and large–size microplastic fragments was 20 and 60% lower, respectively, as compared to beads.

Ziajahromi et al. [59] investigated the chronic and acute effects of microplastics from wastewater treatment plants and PE beads and fibers on freshwater zooplankton, finding that both types of plastics had dose–dependent effects and impacts. The acute contact of both types of plastic had dose–dependent impacts on the endurance. Long–term exposure impacted development and fecundity. Frydkjær et al. [60] examined the absorption, egestion and severe effects of different morphologically shaped PE microplastics in *D. magna*, obtaining different results in absorption and defecation.

Coady et al. [61] assessed the impacts of PE microplastic toxicity on zooplankton for 21 days and found no noticeable impacts on the endurance, fecundity and development. Furthermore, Jemec et al. [62] analyzed the effects of PET textile microfibers on zooplankton after 48 h: the results revealed that the particles raised fatality only in zooplankton not fed with algae, but after feeding, no effects were found. In addition, species–specific trends were observed in some studies, also when comparing organisms with similar ecological behavior. For example, Zebrowski et al. [63] examined the effects of microplastics on various types of zooplankton with respect to superior and inferior competitor: *D. pulex*, *D. magna* and *D. galeata*. The results presented that microplastics’ impacts on the species type were changed. The presence of PS and PE lowered the density of the superior competitor in each of the three pairs, at least partially due to a reduction in the number of gravid females, but not their fecundity.

Schür et al. [64] used passive sampling to investigate fluorescent dye leached from the particles. The results suggested that fluorescence in lipid storage droplets in *Daphnids* caused by a leaching of the dye PEST beads 1000 nm at 2 mg/L would be much stronger, confirming that fluorescence in daphnid tissue occurred due to partitioning of fluorescent dye from the plastic particles to the lipid droplets, helpful in translocation and bioaccumulation.

Finally, an important way forward to understand the role of MPs chemical and morphological features on the effects on zooplankton is the ageing processes of MPs happening in the environment (e.g., degradation due to solar radiation and biofouling). These processes are known to alter chemical and physical properties of MPs in the environment [91,92]. Consequently, they can also reshape the potential exposure pathways and toxicological effects on zooplankton organisms [93]. In addition, ageing of MPs can favor the leaching of (toxic) chemicals present in the fragments, enhancing negative effects on organisms [94,95].

### 3.3. Co–Exposure with Other Stressors

The toxicity of MPs is often analyzed in combination with other stressors in laboratory single–species exposures. Typically, other physical stressors and other different chemicals are added in mixture with MPs when exposing zooplankton.

#### 3.3.1. Physical Stressors

Temperature is a factor in assessing the effects of climate change. For instance, Lyu et al. [85] investigated microplastic impacts and temperature on zooplankton and found adverse effects. The toxicity increased with rising temperature. The increase in temperature caused anomaly in ultrastructure epithelial cells, gene damages which led to oxidative disruptions, energy and reproduction constraints. Microplastic impacts on zooplankton and biomass formation can be exacerbated in a global warming scenario. Additionally, Schür et al. [65] demonstrated the effects of asymmetrical microplastics (<63 mm) and kaolin as a natural substance on the endurance, fecundity and the maturation of zooplankton. Additionally, this study revealed exacerbating fatalities, adverse impacts on fecundity and development. Hoffschröer et al. [96] evaluated the effects of temperature and food supply on the PS (1 μm diameter, concentration of 200 ng/mL) ingestion on *D. magna* and *D. pulex*. An increase in ingestion rates of microplastic beads was observed in the state of low food and elevated temperature.

Conversely, Hiltunen et al. [66] conducted laboratory experiment on microplastic impacts on zooplankton, and no effects on development and fecundity were impacted. Moreover, Guilhermino et al. [67] investigated chronic negative impacts of microplastics on zooplankton with respect to climate change related to population development and fecundity at high water temperature and light intensity, finding that microplastic reduced the population development rate by 27%. Sadler et al. [97] investigated temperature– and clone–dependent effects on zooplankton. It was revealed that high temperature triggers sensitivity and resilience in zooplankton.

#### 3.3.2. Organic Pollutants

The effects of MPs were also tested in combination with other organic chemicals. These types of study were performed to test the hypothesis of the so–called “vector effect” of MPs: MPs can sorb or contain other chemicals which can be transported to the organism after the ingestion of the MPs particles [98,99,100]. These mixed effects were tested on zooplankton using several types of organic chemicals and metals. Rehse et al. [68] investigated the effects of microplastics combined with contaminant bisphenol A (BPA). The microplastic alone did not show adverse impacts, whereas dose–dependent effects were observed in the case of BPA. The integration of BPA and PA posed negative effects with restricted mobilization on zooplankton. Felten et al. [69] examined the individual and combined effects of microplastics with pesticide deltamethrin (DM) pesticide on zooplankton for 21 days. The results revealed that microplastics (10 mg/L) posed adverse effects with the decrease in young zooplankton population, whereas integrated effects of DM and pesticide–caused negative impacts on offspring was observed. Also, the presence of the polyhydroxybutyrate in addition to PE MPs, in turn, increased the population density of *D. magna* in the variants with each of the remaining species, whereas PS and PE lowered the density of the superior competitor in each of the three pairs.

The presence of the polyhydroxybutyrate, in turn, increased the population density of *D. magna* in the variants with each of the two remaining species [63]. Then, Zhang et al. [70] investigated the single and integrated impacts of microplastics and roxithromycin on *D. magna*. It was found that smaller–size microplastics are more dangerous to zooplankton than larger–size ones; 1 μm PS individually or in combination may pose alarming oxidative disruptions compared to 10 μm PS. Then, Wan et al. [101] demonstrated that 15 microcystin–leucine–arginine (MC–LR) microplastics was lethal to zooplankton.

Moreover, Zocchi et al. [71] evaluated a comparative study of different types of glyphosate chemical formulations (glyphosate acid, glyphosate–monoisopropylamine salt and Roundup Gran) and two types of microplastics PE microbeads and PET/PA fibers on *D. magna*. The results suggested noticeably higher mortality in the presence of MPs with all three formulations as compared to the absence of microplastics (i.e., in combination with the PE microbeads or the PET/PA). Even the mortality was slightly increased in combination with Round Gran. In the absence of microplastic, glyphosate–monoisopropylamine salt caused the highest mortality (23.3%), whereas glyphosate (12.5%), acid alone caused the lowest.

#### 3.3.3. Inorganic Chemicals and Nanoparticles

The effects of MPs on zooplankton were also observed in concomitance with the presence of dissolved metals and metal nanoparticles. The combined effects of plastic and copper (Cu) were studied, and it was found that high stress was observed on molting frequency with a single exposure of Cu but not with the mixture of Cu and plastic. Moreover, young ones were more susceptible to Cu with or without microplastic. The exposure after 21 days of microplastic and Cu did not have chronic impacts on zooplankton. Similarly, Dahms et al. [102] stated that zooplankton were sensitive to environmental pollutants. Likewise, Zimmermann et al. [57], Guilhermino et al. [67] and Trotter et al. [103] stated that microplastic restrained zooplanktons’ physical growth and length. While, Martins et al. [72] analyzed the chronic impacts of micropollutants with lithium mixture on zooplankton and found that the contact with mixture up to (0.08 Li + 0.19 MPs mg/L) may decrease the population by 67% and 58%, respectively. Whereas, Yuan et al. [73] examined the negative impacts of microplastics (10 μm and 50 μm) and heavy metals on zooplankton. The results revealed that dose and size of microplastics are important factors with respect to the effects on zooplankton. Enhanced adsorption was observed in smaller microplastics for metal ions. The smaller plastic particle size had more negative impacts due to enhanced bioavailability and adsorption capabilities. In the same way, Lin et al. [74] analyzed acute toxicity and the behavioral effects of plain and functionalized (PS) microplastics on zooplankton. The results of the study suggested that plain microplastics had fatal impacts and negative behavioral impacts on zooplankton, whereas functionalized PS NPs were slighter than plain PS.

Kim et al. [75] analyzed the effects of microplastics on *D. magna* and found an increased immobilization induced by PS–COOH. The toxic effects of PS–COOH were more intense than pristine PS, and it also exacerbated Ni toxicity in co–exposure in comparison to pristine PS. This indicates that the surface properties of MPs play a key role in defining the toxicity in combination with other chemicals. Ma et al. [76] assessed the effects of nanoplastics (ranged from 50 nm to 10 μm) and microplastics (5 μm, 10 μm, 15 μm) on toxicity, bioaccumulation and the environmental fate of phenanthrene in fresh water. The 50 nm particle had no noticeable toxicity or physical injuries, whereas in 14 days’ incubation, the combined effect of NP and phenanthrene prevented the excretion. The toxicity of MPs to *D. magna* relied on size particles showing no bodily disfigurement. Thus, this showed the ease of ingestion and excretion of microplastic beads from the intestines. On the contrary, 50 nm NP at 10 mg/L posed intense harm on thoracopods, necessary for swimming.

Pacheco et al. [77] evaluated the effects of microplastics’ size (1–5 μm) and gold nanoparticles (AuNP) on *D. magna*. The results revealed declined reproduction and immobilized young *Daphnia*. The mixtures caused more toxicity than AuNP and MPs individually. Schrank et al. [78] detected the long–term impacts of flexible and rigid PVC on the shape and life survival traits in *D. magna*. With the contact period of 31 days, rigid PVC and glass beads had no impact on body size. On the contrary, flexible PVC maximized body size and decreased reproduction, while fatality did not increase. As seen from the above, most of the results testing the effect of MPs and other chemicals on zooplankton lead to unclear trends. Both synergistic, antagonistic, and non–significant effects were observed. We highlight here that the interaction of MPs with other chemicals relies on several physiochemical processes, and the adsorption–desorption equilibria should be well established before assessing the further effects on zooplankton [104].

### 3.4. Toward Improved Environmental Relevance: Exposure of MPs to Simplified Communities

Beyond the investigation of the effects of MPs on zooplankton in combination with other stressors, other studies aimed at improving environmental relevance of the ecotoxicological impacts of MPs, exposing simplified communities (e.g., in micro– or mesocosm) to MPs. For example, Setälä et al. [36] revealed for the first time the possibility of microplastic transfer via one trophic level (mesozooplankton) to the advanced level (macrozooplankton) in the food web. In this experiment, food–web transfer was observed by mysid shrimps. The results revealed that microplastics were taken up by various zooplankton species, and their transfer was possible when mysid shrimp (grazers of zooplankton) contained microplastic after feeding on mesozooplankton. Also, the exposure of mysid shrimps occurred both directly and indirectly, indicating multiple pathways of microplastic transfer in the food web.

Small MPs (24 nm) were used as feed in the food chain from algae to zooplankton (*D. magna*) to goldfish (*Carassius*), with visible effects on the feeding behavior and metabolic effects such as weight loss. The fish in contact with MPs doubled the consumption time for the same amount of zooplankton as compared to control fish [105]. Verification of trophic transfer of microplastics emerges from the quantification of MPs in the organisms collected in the field, their ecological predators, and discreet feeding experiments that led to a prototype of microplastic transfer via the fabricated food chain [34,106,107].

Wang et al. [79] studied instead the impacts of PS MPs on morphological structure, fecundity of zooplankton under the predation risk of zooplanktivorous fish *Rhodeus ocellatus*. It was observed that the defense mechanism was compromised. However, swimming movement, pulse rate and thoracic appendages were not impacted. Smaller particles weakened the defense mechanism more than larger ones.

Furthermore, Mavrianos et al. [80] analyzed microplastic impacts individually and integrated with microparasites *Metschnikowia* on zooplankton health. It was revealed that a shortened life period and reproduction were observed. Zooplankton’s exposure to microplastics for almost 5 days was lethal. Moreover, for all the microplastic concentrations, this led to a reduction in fecundity.

Further, Elizalde–Velázquez et al. [81] investigated the decontamination of two concentrations of 6 mm PS MPs on *Daphnia* and on the fish species *Pimephales promelas*. The presence of microplastic remained for 5 days in the gastrointestinal (GI) tract of both species, and after 96 h exposure, microplastics were excreted. This study revealed that the presence of food impacted the gut cleaning of *Daphnia*, and no transfer of microplastic entered in tissues and organs via the GI tract.

Yıldız et al. [82] examined the effects of MPs on a lake ecosystem using in situ experiments: model food web with zooplankton as herbivores, odonate larvae as predators and chironomid larvae as detritivores for seven weeks were exposed to MPs. The results showed that MPs uptake for the zooplankton was low and constricted to bigger–size *Daphnia*, leading to biomass reduction. Whereas, biomass for other zooplankton was not reduced. The existence of MPs in the fecal pallets of odonate larvae that consumed zooplankton was evidence of MPs trophic transfer.

### 3.5. Future Trends and Research Gaps in the Exposure of Zooplankton to MPs

**Figure 3:**
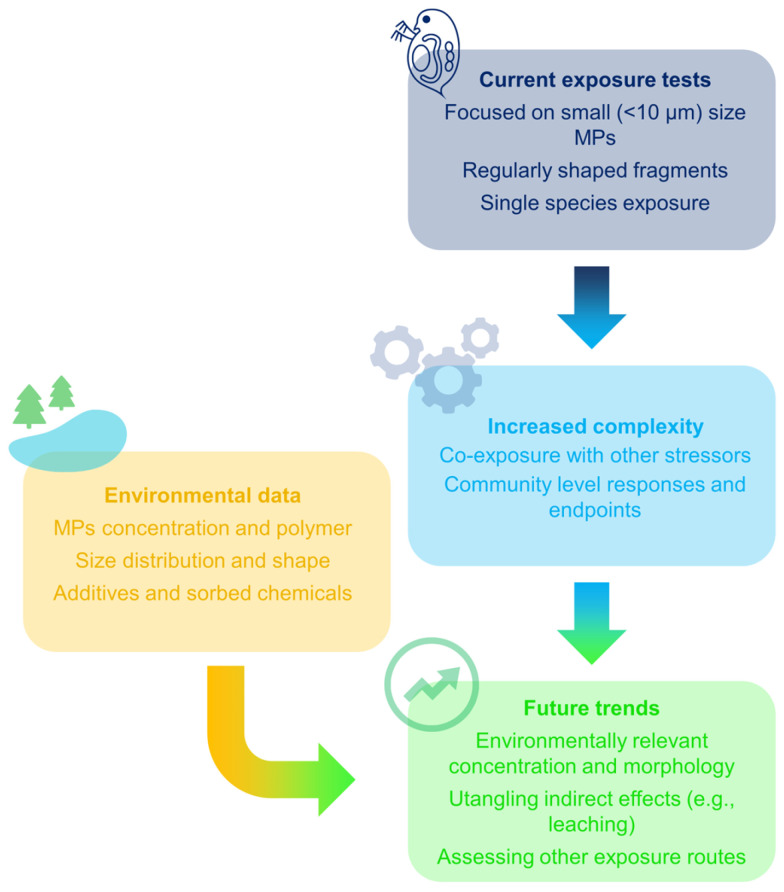
Scheme of the current and potential future trends in the analysis of zooplankton exposure to MPs, highlighting the role of environmental studies in tailoring the exposure conditions.

The trends summarized here show a focus on the hazard assessment of MPs to zooplankton in freshwater environments. However, while some first attempts to understand more complex, community–level responses were recently investigated, a main link with environmentally relevant conditions is still missing. Environmental data can, in fact, provide guidance on the environmental boundary conditions in order to tune, at best, ecotoxicological tests (Figure 3).

However, a key role is observed to be played by the particle properties of MPs fragments. The morphological characteristics frequently used in bioaccumulation study include spherical particles and pristine pellets, which limitedly represent plastics present in aquatic ecosystems. Besides, the plastic that is used is imported by manufacturers and is not damaged or impacted by microbial contamination, which could lead to an increased risk of MPs pollution.

These unclear trends indicate that single–species tests may lead to overestimation or underestimation of the risk, depending on the species sensitivity and the exposure route. In fact, plastic may affect zooplankton in several (and often indirect) ways. Moreover, beyond ingestion, MPs may induce negative effects due to adsorption on the organisms [108]. Therefore, we recommend a more detailed investigation of the exposure route and of the particle properties when investigating the potential risks of MPs to zooplankton in lab studies.

We suggest therefore the investigation of environmentally relevant particles: we suggest comparing the effects of pristine particles with plastic obtained from real–world objects, containing additives and presenting irregular shapes. We would also support future studies investigating the effects of the particle uptake route, egestion and other potential indirect effects. In fact, plastic may affect zooplankton in several (and often indirect) ways: for instance, MPs may induce negative effects due to adsorption on the organisms [108]. Therefore, we recommend a more detailed investigation of the exposure route and of the particle properties when investigating the potential risks of MPs to zooplankton in lab studies.

Finally, we recommend investigating more complex systems to assess the ecological effects of MPs exposure in freshwater bodies. In fact, MPs revealed several negative implications to fresh water when analyzing the effects at an ecosystem scale. For example, Pan et al. [83] analyzed the impacts of PE microplastics on zooplankton’s functioning, feeding and the trophic decline impacts on the food chain amid its sustenance and perseverance in Dianchi Lake, China. The experiment revealed that microplastics lowered the grazing rates with decline of heart rate and jumping capability. Chronic contact of microplastic had negative impacts on its sustenance and perseverance in grazing capabilities, with fecundity reduction and amplified predation risk.

## 4. Studies on Zooplankton and Microplastics in Freshwater Lakes

Field studies monitoring the environmental concentration of MPs in zooplankton are scant, especially considering freshwater lakes. In this review, we focused on the studies reporting MPs in environmental samples of zooplankton from lacustrine ecosystems (the results reported in this paragraph are summarized in Table 2). We would clarify here that while these studies reported MPs of various size and polymer connected to zooplankton samples, the effective ingestion or other exposure route were unfortunately impossible to discriminate.

### 4.1. Current Knowledge of Environmental Conditions

Pazos et al. [109] investigated the impacts of microplastic on zooplankton with respect to morphological structure and dimension in the freshwater body Río de la Plata estuary, South America. Lusher et al. [110] detected microplastic in zooplankton from Lake Mjøsa and found that microplastics were found in all samples, with 97% of the occurred particles having size <1 mm. Rubber was commonly found in zooplankton. Alfonso et al. [115] published a review paper and highlighted zooplankton as a tool for MPs assessment in water associated with particle ingestion in zooplankton. The author claimed that ingestible MPs size was below 50 μm. They also observed irregular fragments composed of variable polymer types in the environmental samples reported. Pastorino et al. [111] investigated biotic (zooplankton, fish and tadpoles) and abiotic samples (water and sediment) and did not detect MPs in zooplankton and water, whereas MPs were present in sediments. Although, Wu et al. [116] collected several studies and published a review paper on the microplastics impacts on zooplankton in freshwater bodies of China and revealed that Poyang Lake China had the highest concentration of 34 items/L microplastics and has adverse effects on the digestive tract of zooplankton, particularly on grazing, fecundity and development. Franzellitti et al. [117] summarized a review paper of MPs distribution and effects regarding potential changes at the molecular, cellular and systemic levels on a wide range of aquatic organisms, which states that the effects of MPs, particularly on crustacean *D. magna* uptake of PET textile fibers, escalated mortality irrespective of the feeding pattern [62]. Additionally, another screening study on uptake and the effects of MPs on *D. magna* suggested that zooplankton crustaceans can uptake various MPs without causing highly acutely hazardous effects on them [51].

Bowszys et al. [118] published a review paper of 85 lakes of over 500,000 European lakes (>0.01 km^2^) in the form of a review study by using the keywords microplastic(s), plastic, lake(s), fresh water, Europe, zooplankton and fish. They quantified MPs size with a size >5 mm to identify the most urgent areas of research that are required in the field of microplastic pollution. The results of the review suggested limited data on microplastic pollution <300 μm. There is ambiguity due to a knowledge gap since some articles indicate that the microplastic quantity may increase intensely if the focus is placed on smaller particles. Similarly, limited data are found on the fate of microplastics in the water column and the influence they have on lake zooplankton. This study states that there is a lack of substantial evidence of microplastic ingestion by zooplankton in a natural environment but highlights the clear possibility of trophic transfer of microplastic in lake food webs through a diverse range of aquatic organisms.

Da Silva et al. [112] examined the effect of varied–size microplastic particles on zooplankton communities from a lake located in the Upper Paraná River floodplain, Brazil. It was demonstrated that MPs particles may have serious impacts on the trophic web. It was revealed that most MPs ingestion effects come from the base levels of the food chain.

Klasios and Tseng et al. [113] quantified and characterized microplastics subsurface water and zooplankton from eight lakes in BC, Canada, to understand the microplastic entrance in the food web. The results suggested that fibers were predominant in all lakes. Further, Raman spectroscopy determined PEST as the dominant polymer in zooplankton and water. Moreover, zooplankton consume shorter microplastic than body size.

Rajeswari et al. [114] investigated microplastic pollution in Kolavai Lake, Tamil Nadu, India, and highlighted the microplastic–to–zooplankton ratio and its severe impact on the environment’s food chain. Further chemical and morphological characteristics were studied using Fourier transform infrared spectroscopy (FTIR) and SEM analysis. The results revealed the intense abundance of microplastic as a consequence of human activities. The microplastic–to–zooplankton ratio was found to be in the range from 0.05 to 0.74. The results support that microplastic may have detrimental impacts due to infiltration in the food web.

### 4.2. Future Steps for Environmental Monitoring of Zooplankton–MPs Interaction

As a matter of fact, this review highlighted a dire need to conduct more field study considering that microplastics exposure to zooplankton in lab is higher than in the field with entirely different conditions. Further, the review represents an increasing interest in the field of microplastics and zooplankton as it included many publications (1457) related to this field. Most of the studies highlight a gap between the real–world situation and experimental conditions since the information on the level of contamination related to zooplankton in environmental conditions is limited. As an example, the most frequently analyzed polymer in ecotoxicological studies is observed to be PS, while other polymers such as PP are rarely tested (Figure 2). This is in contrast with several environmental studies in freshwater bodies, which reported a dominance of PP and PE as the most abundant plastic debris and MPs [119,120]. While the number of reports assessing the content of MPs in the water column and sediments of lake is continuously increasing [21,119], this review discovered the insufficient information related to the effects of microplastics on populations and ecosystems, indicating a need for more studies in this field (Figure 3). The trends represent an increasing interest in the interactivity between microplastics and plankton, but due to knowledge gaps regarding the comprehension of the effects on populations and ecosystems, the deviation between laboratory and field conditions implies the need for more field studies and systematic methodologies [121].

In this sense, environmental monitoring programs aimed at understanding the interaction between MPs and zooplankton organisms may be the key to unfolding novel and relevant ecotoxicological tests (Figure 3). Investigation of this kind will unravel the relevant concentrations and the polymer type, size distribution and particle features of common MPs in freshwater bodies, providing guidance for future exposure and effect tests in laboratory. A main hindrance in this process is the need for data and methods harmonization, which is unfortunately a known issue in MPs research [119,122].

## 5. Conclusions and Research Outlook

The current review highlights the impacts of microplastics in lakes with respect to zooplankton as a practical assessment tool to be an indicator of pollution in aquatic ecosystems. This study helps to direct the environmental scientists to strive to fill the indicated knowledge gaps in the field of microplastic pollution and freshwater environments to establish improved standards for microplastic pollution prevention worldwide. Based on the findings of this review, it is a prerequisite to study zooplankton dynamics to assess the risk assessment for the aquatic environment. Further research should be considered to resolve the issue of trophic transfer of microplastics in field sampling organisms and their predators and simulation of trophic transfer experiments in laboratory feeding. It is important to develop multilevel trophic investigations based on the top predators. The research in this regard is scarce: it is more focused on the secondary food chain and laboratory. Still, aspects such as the characteristic of links between MPs and other contaminants and freshwater microplastic contamination, microplastic concentration extent, standard protocols, microplastic fate, and interaction with other pollutants needs more research to have an accurate risk assessment of and preventive measures for microplastic pollution.

## Figures and Tables

**Table 1:** Summary of the main results of laboratory–based ecotoxicological studies.

Zooplankton Type	MPs Concentration in Zooplankton	Affected Endpoint	MPs Size	MPs Type Morphology (Fiber, Pallets, Fragments, etc.)	Exposure Assessment Results	References
*D magna*	PS–NPs; 0.05 and 0.5 μg/mL	Significant changes in energy reserves. No alteration of swimming activity.	50 nm	PS spherical nanoplastics	Gene associated with oxidative stress response occurred within contact of 21 days.	[45]
*D. magna*	0.125, 1.25 and 12.5 μg/mL)	Enhanced body size and swimming activity	1 μm and 10 μm	PS beads	No noticeable risk.	[46]
*D. magna*	2, 4 and 8 mg/L	Oxidative production declined.	1.25 µm	PS microbeads	Long–term contact of microplastics impacted the oxidative protection mechanism at 8 mg/L. Microplastics depuration from cells escalated the microplastics toxicity.	[47]
*D. magna*	1.46 × 102 mg/L	Impacted fatality not reproduction.	2 μm	PS	Single concentration of MP was consumed rapidly in large quantities. Daphnia did not consume MP in the presence of algae	[48]
*D. magna*	6 MPs/mL	No impact.	5 μm	white, green, orange and red PS round–shaped microbeads	Daphnids were unable to differentiate between colored MPs and algae. The phenomenon was stopped on the third day due to accumulation of MPs.	[49]
*D. magna*	0.5, 1, 2 and 4 mg/L)	Digestive tract damage. 2 and 4 mg/L: impeded survival.	500 nm	PS	MPs 500 nm constrained antioxidant processes and interfered with energy metabolic pathways.	[50]
*D. magna*	100 mg/L	No impacts.	300 μm, 120 μm and 20 μm	facial cleanser products, 1 plastic bag and 1 textile fleece	Small MPs particles were not hazardous.	[51]
*D. magna*	12.5–400 mg/L	Increased immobilization.	1 and 100 µm	PE spherical particles	Easy ingestion of small microplastics. 1 µm ingestion caused immobilization increasing with dose and time after 96 h. No ingestion with 100 µm particles.	[52]
Rotifers	0, 0.1, 1, 10 and 20 μg/mL)	10 μg/mL: oxidative damage to cells and membrane.	0.05, 0.5 and 6 μm	PS microbeads	Microbeads intake occurred easily. Microbeads scattered in the digestive tract.	[53]
rotifer *Brachionus plicatilis*	(5 μg/mL, 10 μg/mL, 20 μg/mL and 50 μg/mL)	>10 μg/mL: reduced lifespan, development, reproduction.	50, 100, 500 nm	PS pallets	Small–size MPs posed negative impacts. Large particles were not harmful.	[54]
*D. magna*	2 mg/L.	Increased toxicity via translocation.	20 nm and 1000 nm	PS microbeads	Fast excretion with larger beads up to 90% in 4 h as compared to small–size beads in 4 h up to 40%. Ingestion of small–size particles was equal to/greater than large–size beads.	[55]
Monogonont Rotifer (*Brachionus koreanus*)	0.1, 1, 10 and 20 μg/mL.	Declined development, fertility, reproduction and lifetime.	0.05, 0.5 and 6 μm	PS microbeads	Small microbeads are more toxic than large ones. Adverse effects of microplastics are size–dependent. Large microbeads depurated easily.	[56]
*D. magna*	10, 50, 100 and 500 mg/L.	Reduced reproduction, survival traits and offspring.	2.0–60 µm and 8.0–240 µm	PVC, PUR and PLA microplastics irregular particles	At 10 mg/L, survival was impacted. Microplastic declined the propagation and reduced the number of offspring from 101 to 34 at 100 mg/L and to 0 at 500 mg/L.	[57]
*D. magna*	5 mg/L	Decreased number of juveniles, body length and survival traits.	small– and large–sized MPs fragments (17.23 and 34.43 µm) MPs beads (39.54 µm).	PE MPs fragments (large irregular) and beads (small spherical regular).	Resilience to small– and large–sized MPs fragments was 20 and 60%, respectively, on exposure as compared to MPs beads (90%), i.e., less upon contact with MPs.	[58]
*Ceriodaphnia dubia*	0.5 to 16 mg/L of PE beads and 0.125 to 4 mg/L of PEST fibers.	Reduced reproduction. Increased fatality.	1–4 μm PE microplastic 116 μm beads	PEST fibers and PE spherical beads	Fibers caused a 50% decline in propagation. Fibers affect zooplankton more negatively than beads. 100% fatality at 4 mg/L for PEST fibers and 8 mg/L for PE beads.	[59]
*D. magna*	0.0001–10 g/L	Increased immobility.	beads (10–106 μm) and fragments (10–75 μm)	PE microplastic, two types: regular round–shaped beads and irregular–shaped fragments.	Regular–shaped beads caused immobility after 48 h at 5 g/L, i.e., 50% less than irregular fragments. Slower depuration of irregular fragments.	[60]
*D. magna*	9.2 and 69 mg	Reproduction, survival and development were unaffected.	144 and 543 nm	Ethylene acrylic acid copolymer	No impact on development, fecundity or resilience in 21 days.	[61]
*D. magna*	12.5–100 mg/L	Increased fatality.	length range: 62–1400 µm, width 31–528 µm, thickness 1–21.5 µm	PET regular textile microfibers	Ingestion of 300 µm occurred beside huge fibers around 1400 µm. Fatality escalated with fibers after 48 h only with zooplankton not fed with algae.	[62]
Superior and inferior competitor: *D. pulex* and *D. magna*, *D. magna* and *D. galeata*, *D. pulex* and *D. galeata*	0.2 mg/L	Reduced female population.	PS 23.3 μm and PE 23.0 μm	PS and PE	No. of particles stored: *D. pulex* stored PS (46.09) more than PE (2.1) as compared to *D. galeata.* Higher accumulation of PS particles, i.e., (44.30) compared to PE (19.73), irrespective of species.	[63]
*D. magna*	2 mg/L	Inflammation and bioaccumulation.	20 and 1000 nm	PS beads	Microplastic leach the fluorescent dye. Both 20 nm and 1000 nm were visible in the gut. Translocation poses internal damage and bioaccumulation.	[64]
*D. magna*	10,000 and 2000 particles/ mL	Decreased reproduction, development and survival mechanism.	<63 µm	Irregular PS particle	Irregular MPs cause more toxicity than Kaolin.	[65]
*Daphnia*	0.03 mg C/L	Reduced reproduction, growth and survival.	1.2–40 μm	PET, PS, tray and toy nbrick (acrylonitrile butadiene styrene)	Declied endurance almost (81% to 21%), young ones’ size (1.8 mm to 1.0 mm), mature size (2.7 mm to 1.1 mm), and propagation (13 offspring per surviving adult to 0).	[66]
*D. magna*	0.04, 0.09, 0.19 mg/L	Reduced population.	1–5 μm diameter	Polymer microspheres.	0.19 mg/L reduced the population dynamics up to 38% at 20 °C and 59% at 25 °C. MPs at higher temperature decrease population more than lower temperature.	[67]
*D. magna*	200 mg/L	Decreased immobilization.	15–20 μm	Irregular–shaped PA particles	No impact of PA particles alone. Combined effect of PA and BPA led to decreased immobilization.	[68]
*D magna*	0, 1, 10 mg/L	Delayed fertility, reduced neonates, and survival mechanism.	1–4 μm	PE spherical particle	MPs 10 mg/L declined the young zooplankton. Combination of Deltamethrin and MPs declined resilience, female population by 51.1% and 46% offspring.	[69]
*D. magna*	0.1 mg/L	Increased immobilization and physical damage.	1 and 10 μm	PS particles	MPs and roxithromycin (ROX) both alone and individually trigger biological responses. 48 h exposure to PS (0.1 mg/L) or ROX (0.01 mg/L) alone led oxidative stresses.	[70]
*D. magna*	2.5 mg/L	Increased fatality.	length of 10 μm and width of 2 μm	PE microbeads and (PET/PA fibers)	PE escalated fatality as compared to PET/PA, but at 168 h fatality values were close, i.e., 38.3% and 31.7%. Fatality escalated PET/PA+ glyphosate acid (by 17.5% after 168 h).	[71]
*D. magna*	0.02 Li + 0.04 MP, 0.04 Li + 0.09 MP mg/L, 0.08 Li + 0.19 MP mg/L	Increased fatality rate and reduced population growth.	1–5 µm	fluorescent plastic microspheres	(0.08 Li + 0.19 MP mg/L) declined the population dynamics up to 67% and 58%, respectively. Declined the physical growth by 20% and 40%, respectively.	[72]
*D. magna*	lower MPs concentrations (0.01–10 mg/L) and higher MPs concentrations (10–1000 mg/L)	Increased immobilization and physical disfigurement.	10 μm and 50 μm	PS beads	Small microplastics, individually or in groups, create more immobilization and physical deformation as compared to large at low concentrations. Small microplastics have enhanced adsorption capacities on metals as compared to large.	[73]
*D. magna*	concentrations of plain PS, PS–COOH, PS–n–NH_2_ and PS–p–NH_2_ in exposure suspensions were set at a range of 0–75 mg/L, 0–70 mg/L, 0–40 mg/L and 0–100 mg/L, respectively	Increased fatality.	100 nm, 50–100 nm and 300 nm	PS	Pure microplastic was more toxic than functionalized microplastic. Functionalized PS–p–NH_2_ caused no immobilization.	[74]
*D. magna*	1, 5, 10, 20 and 30 mg/L	Increased immobility and toxicity.	PS: 201.5 and PS–COOH 191.3 nm	carboxyl group (PS–COOH) and PS	Toxic effects of PS–COOH were higher than PS. Nickel (Ni) with PS–COOH was higher in toxicity as compared to a mixture of (Ni) with PS.	[75]
*D. magna*	(1–50) mg/L up to 100 mg/L	10 mg/L: extreme physical and swimming impediment.	50 nm to 500 nm nanoplastics and (5 μm, 10 μm, 15 μm) microplastic.	Beads	Integration of nanoplastics and phenanthrene prevented the excretion of particles. MPs did not show any bodily disfigurement.	[76]
*D. magna*	12 mg/L	Increased fatality, decreased reproduction, and immobilization.	1–5 μm	Not mentioned	Mixtures caused more toxicity than AuNP and MPs individually.	[77]
*D. magna*	2.67 μg/L	Increased body size. Decreased reproduction.	100–150 μm	PVC	Rigid PVC and glass beads had no impact on body size. Flexible PVC maximized body size and decreased reproduction. Fatality did not increase.	[78]
*D. magna*	2 and 6 mg/L	Reduced reproduction and defense mechanism.	0.7 μm–3 μm	PS spherical plastic	MPs 0.7 μm damaged the protection mechanism. Small–size MPs are more dangerous than large ones.	[79]
*Daphnia galeata*	5 and 20 mg/L	Reduced fertility and spore production.	≤100 nm	PS spherical particles	Life duration shortened in 12 days. High concentration caused mortality in 5 days.	[80]
*D. magna*	20–2000 mg/L	Negligible bioaccumulation. No impacts on tissue.	6 µm	PS microsphere rounded shape	Microplastics were found in the GI tract up to 5 days’ contact. Microplastics were excreted between 72 and 96 h, majorly influenced by the food existence. Low bioconcentration caused fast excretion.	[81]
*Daphnia*	2 mg/L for the water column (PE with fluorescent)	Lower *Daphnia* biomass	200 μm	PE, PP, PS, PVC, PA and PET	Biomass for other zooplankton was not reduced. Evidence of MPs trophic transfer was shown.	[82]
*D. magna*	0, 5, 40 and 160 mg/L	Retardation in fertility and reproduction.	<70 μm	PE particles	MPs may impact the resilience and behavioral–affiliated changes. MPs 40 mg/L hampered two days reproduction and particular female neonates.	[83]
*D. magna*	1 mg/L, 0.5 mg/L and 0.1 mg/L	No impact on reproduction.	2 μm and 100 nm	PS spherical beads	Declined feed rate up to 21%. Slow evacuation caused declined feeding rate.	[84]
*D. magna*	2 mg/L	Increased fatality Reduced reproduction and population dynamics.	1.25 μm	PS particles	MPs toxicity and energy decline were more at higher temperature, i.e., 30 °C compared to 20 °C.	[85]
*D. magna*	(0.4 and 9 μgC/mL) and MPs	Increased fatality and reproduction.	Beads (1–5 μm) of fluorescent PE.	PMP: PE spherical beads and SMP; PE irregular beads	Secondary microplastics (SMPS) escalated fatality and declined propagation at high MPs levels. Doubled gut passage time with SMP. SMPS have more negative impacts.	[86]
*D. magna*	200 ng/mL or 360,000 particles/mL	Zooplankton did not show harmful impacts.	1 μm	PS beads	High temperature in natural environment may be harmful for *Daphnia*	[87]

**Table 2:** Summary of the studies analyzing MPs related to zooplankton in freshwater lakes.

Water Body (Lake)	Location	Zooplankton Type	MPs Concentration (m^3^) in L	MPs Size	MPs Type Morphology	MPs (Source)	Result	References
Lake Taihu	China	*crustacean*, *D. magna*	Not mentioned	200 μm	PE and PP particles	Not mentioned	A prolonged period escalated adsorption by 25.1% and 6.5%. Later, desorption posed extreme risks to zooplankton.	[101]
Río de la Plata estuary (South America)	South America	*rotifers*, *copepods*, *cyclopoida* and *nauplius larvae*	164 and 114 MPs m^3^.	>500 and ≤1000 μm	fibers and fragments	urbanized sites, sewage discharges	Fibers were present in all samples. All zooplankton (mainly mesozooplankton) contained MPs.	[109]
Lake Mjøsa	Norway	Zooplankton	0.001–0.06	Fragments (294 μm to 153 μm)	Rubber, PE, PS, PVC, acrylic. Fibers and fragments	Not mentioned	Fibers and fragments were present in all samples.	[110]
Lake Balma	Italy	Not mentioned	Not present	Not mentioned	Not mentioned	Not mentioned	MPs were not found in zooplankton.	[111]
Garças Lagoon	Brazil	*Cladoceran* and *copepods*	30 ind./L, 64 ind./L	0.75 μm, 1.0 μm and 3.0 μm	Beads	Not mentioned	Highest ingested 0.75 μm and 1.0 μm MPs particles. Evidence of microplastic transfer.	[112]
8 lakes in BC, Canada	Canada	*Copepod*, *Daphnia*	0.01 ± 0.011 microplastics per copepod and 0.02 ± 0.014 microplastics per *Daphnia*	Not mentioned	PEST fibers and PET films and fragments	Recreational activities	PEST was dominant in zooplankton. Zooplankton consume shorter microplastic than body size.	[113]
Kolavai Lake	India	*Rotifera*, *nauplii* and *Cyclopoida*, *Cladocera* and *Calanoida*	6.1 ± 2.5 particles/L	>0.3 mm	Fibers and fragments: PE, high–density polyethylene (HDPE) and PP	Road and solid waste pollution	microplastic–to–zooplankton ratio 0.05 to 0.74 MPs have detrimental impacts due to infiltration in the food web.	[114]

## Data Availability

Not applicable.
